# The Prognostic Significance of C-Reactive Protein to Albumin Ratio in Patients With Severe Fever With Thrombocytopenia Syndrome

**DOI:** 10.3389/fmed.2022.879982

**Published:** 2022-04-29

**Authors:** Xiaozhou Yang, Huimin Yin, Congshu Xiao, Rongkuan Li, Yu Liu

**Affiliations:** Department of Infectious Diseases, The Second Affiliated Hospital of Dalian Medical University, Dalian, China

**Keywords:** inflammation-based indexes, C-reactive protein, albumin, SFTS, disease prognosis assessment

## Abstract

**Background:**

Severe fever with thrombocytopenia syndrome (SFTS) is an emerging infectious disease with the high case-fatality rate, lacking effective therapies and vaccines. Inflammation-based indexes have been widely used to predict the prognosis of patients with cancers and some inflammatory diseases. In our study, we aim to explore the predictive value of the inflammation-based indexes in SFTS patients.

**Methods:**

We retrospectively analyzed 82 patients diagnosed with SFTS. The inflammation-based indexes, including neutrophil-to-lymphocyte ratio (NLR), monocyte-to-lymphocyte ratio (MLR), platelet-to-lymphocyte ratio (PLR), systemic immune-inflammation index (SII), systemic inflammation response index (SIRI), aggregate index of systemic inflammation (AISI) and C-reactive protein to albumin ratio (CAR), were compared between the survival and death patients. Receiver operating characteristic (ROC) curves were used to compare the predictive ability of MLR, AISI, and CAR. The survival analysis was based on the Kaplan–Meier (KM) method. Multivariate logistic regression analysis was used to analyze the independent risk factors of poor prognosis in patients with SFTS.

**Results:**

The CAR is higher in the death group while MLR and AISI were higher in the survival group. The ROC curve analysis indicated CAR exhibited more predictive value than the other indexes and the optimal cut-off value of CAR was equal to or greater than 0.14. KM survival curve showed that higher CAR was significantly correlated to the lower overall survival in SFTS patients. Multivariate logistic regression analysis indicated that CAR was an independent risk factor for poor prognosis in patients with SFTS.

**Conclusion:**

The CAR is an independent risk factor for death in patients with SFTS and could predict the poor prognosis of SFTS patients. It could be used as a biomarker to help physicians to monitor and treat patients more aggressively to improve clinical prognosis.

## Introduction

Severe fever with thrombocytopenia syndrome (SFTS), an emerging infectious disease, is caused by a novel member of phlebovirus called SFTS virus (SFTSV) which is classified into the Bunyaviridae family (1). The SFTS was first reported in China in 2009 and subsequently reported in Vietnam, Japan, South Korea and other Asian countries (1–4). This disease is mainly transmitted by tick bite from *Haemaphysalis longicornis*. The SFTS antibody seroprevalence presented in mammal including wild boars, sheep, cattle, cats, and dogs (5–8). A few studies had provided evidence for the direct cat-to-human transmission of the virus (9, 10). In addition, transmission through close contact with blood or body fluid from infected patients is also reported (11–13). The SFTSV infection is characterized by abrupt onset of fever, thrombocytopenia, leukopenia and other non-specific clinical manifestations with a reported case fatality ratio ranging from 2.5 to 47% in different areas (1, 14–16). The mortality rate is showing a decreased trend due to more cases identified by the viral RNA or SFTSV specific antibodies (17).

Pathogenesis of SFTS is not well described. A common pathogenic feature of bunyaviruses is their ability to inhibit the host immune response. Currently, the main therapeutic plan is symptomatic and supportive therapy. Ribavirin as an antiviral therapy have been applied to SFTS patients but the efficacy remains limited (18). Favipiravir is a new anti-influenza drug approved for human use in Japan (19) and has showed efficacy for the prevention and treatment of SFTSV infection in animal models (20, 21). Favipiravir treatment lowered the case fatality rate of patients with SFTS by around 10% compared with those received supportive therapy in a few clinical trials (22–24). However, the sample size is limited and the side effect is still unclear. The disease is still an important public health problem. The present studies mainly focused on describing the clinical features and laboratory results. Therefore, it is necessary to discover prognostic biomarkers that can contribute to clinical decision-making and help to individualize treatments for SFTS patients.

Regular laboratory tests for the assessment of inflammatory status and organ function are very useful in the early phase diagnosis of many diseases. The complete blood count could provide information including different cell types and morphological parameters. In addition, it is very easy and inexpensive to perform. The ratios derived from the complete blood count such as neutrophil-to-lymphocyte ratio (NLR), monocyte-to-lymphocyte ratio (MLR), and platelet-to-lymphocyte ratio (PLR) have been used as inflammation indexes to evaluate the progression, risk and prognosis of inflammatory diseases (25–28). Other indexes including three or more blood count values, systemic immune-inflammation index (SII), systemic inflammation response index (SIRI) and aggregate index of systemic inflammation (AISI) are considered to reflect the immune and inflammatory state, and have been observed related to risk and mortality of various cancers (29, 30), autoimmune diseases (31) and infectious diseases (32). The C-reactive protein (CRP) is an acute phase protein produced in the liver and is utilized as a marker of infection and inflammatory processes. Albumin (ALB) is synthesized by the liver. Hypoalbuminemia is an indicator of liver dysfunction, malnutrition, systemic inflammation and some other diseases (33). The combined indexes CAR (CRP/ALB) and prognostic nutritional index (PNI) based on the CRP, ALB and peripheral blood lymphocyte count have been used to predict the poor outcome in malignant and non-malignant diseases (34–38). The inflammation and malnutrition are ubiquity in SFTS patients, but few studies have investigated their prognostic value.

In our study, we aimed to investigate the association of these indexes including NLR, PLR, MLR, AISI, SII, SIRI, PNI, and CAR with the SFTS, hoping to find a valuable marker for predicting the prognosis of SFTS.

## Materials and Methods

### The Enrolled Patients

A total of 82 patients admitted from July 2016 to November 2021 in the Second Affiliated Hospital of Dalian Medical University were enrolled in this retrospective study. All the patients were confirmed by detecting the SFTSV RNA in the blood samples collected at admission. The patients were then divided into 2 groups depending on their clinical outcomes, the death group with 32 patients and survival group with 50 patients. This study was approved by the research ethics committee of the Second Affiliated Hospital of Dalian Medical University and performed in accordance with the principles of the Declaration of Helsinki. The information of these patients was anonymous, non-identifiable and maintained with confidentiality. Written informed consent from each patient was waived because of the nature of observational retrospective study.

### Data Collection

The demographic data and clinical outcomes of patients were collected from the electronic medical system. The laboratory tests at admission including the CRP, liver function, kidney function, heart function, and routine blood parameters were collected and evaluated. All laboratory data were tested in the same laboratory using standardization and certification procedures.

The indexes were calculated according to the following equations: NLR = neutrophil/lymphocyte counts; PLR = platelet/lymphocyte counts; MLR = monocytes/lymphocyte counts; CAR = CRP/ALB ratio; SIRI = neutrophil × monocyte to lymphocyte ratio; AISI = (neutrophils × monocytes × platelets)/ lymphocytes; PNI = albumin (g/L) + 5 × total lymphocyte counts (10^9^/L); SII = platelet × neutrophil/lymphocyte counts.

### Statistical Analysis

All the data analysis was performed using SPSS 23.0 software (SPSS Inc., Chicago, IL, United States). The continuous variables were expressed as median (interquartile range, IQR) and compared by Mann-Whitney *U* test. The categorical variables were expressed as number (%) and compared by chi-square test or Fisher’s exact. Receiver operating characteristic (ROC) curve analyses were performed to find the optimal cut-off values, maximizing sensitivity and specificity of biomarkers for distinguishing the death and survival SFTS patients based on best Youden index (sensitivity + specificity-1). Binary logistic regression analysis was performed to identify the independent risk factors for mortality of SFTS, variables with *p* value < 0.05 were included in the multivariate logistic regression analysis by the method of forward stepwise selection based on the likelihood ratio. Survival analysis was performed using the Kaplan-Meier (KM) curve based on the log-rank test. A two-sided *p* value of < 0.05 was considered as statistically significant.

## Results

### Baseline Characteristics of the Patients

**FIGURE 1:**
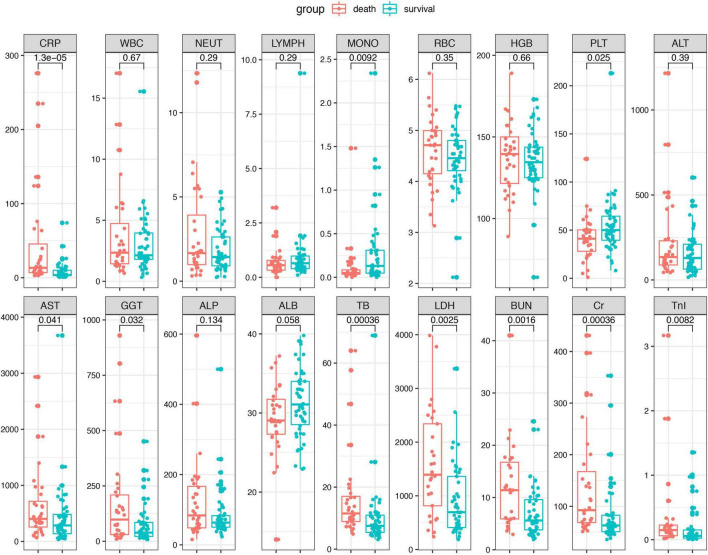
The laboratory parameters were compared between the death patients and survival patients. Statistical significance was calculated by Mann–Whitney *U* test. Data are presented as median (IQR). WBC, white blood cell; LYMPH, lymphocyte; NEUT, neutrophil; MONO, monocyte; ALT, alanine aminotransferase; AST, aspartate aminotransferase; GGT, gamma-glutamyl transpeptidase; ALP, alkaline phosphatase; ALB, albumin; LDH, lactate dehydrogenase; TB, total bilirubin; BUN, blood urea nitrogen; Cr, creatinine; TnI, cardiac troponin I.

A total of 82 patients with confirmed SFTSV detection were enrolled in this study. During hospitalization, 32 patients progressed to death. The clinical characteristics and outcomes of patients between the death group and survival group were summarized in Table 1 and Figure 1. There were 40 male patients and 42 female patients. The median age of the studied patients was 65 years. There was no significant difference in age and gender between the death and survival group. The levels of CRP, AST, GGT, LDH, TB, BUN, CR, and TnI were significant higher in the death group than in the survival group. And compared to the survival group, the death patients had significantly lower monocyte counts and platelet counts. The remaining biomarkers displayed no differences between the two groups.

**TABLE 1:** Baseline characteristics of patients between survival group and death group.

	Normal range	Survival (*n* = 50)	Death (*n* = 32)	*p* value
Age		62 (55–71)	67 (56.5–73.8)	0.354
Gender, male (%)		24 (48)	16 (50)	0.86
Hospital stay		9 (6–12)	4 (2–5.8)	<0.001
CRP, mg/L	0–5	3.7 (2.9–10.5)	13.4 (6.8–57.7)	<0.001
White blood cell count, × 10^9^/L	3.50–9.50	2.1 (1.8–4.0)	2.34 (1.3–5.6)	0.672
Neutrophil count, × 10^9^/L	1.80–6.30	1.4 (0.9–2.7)	1.67 (1.0–4.7)	0.287
Lymphocyte count, × 10^9^/L	1.10–3.20	0.65 (0.39–1)	0.56 (0.3–0.83)	0.287
Monocyte count, × 10^9^/L	0.10–0.60	0.13 (0.04–0.31)	0.05 (0.03–0.09)	0.009
Red blood cell count, × 10^9^/L	3.80–5.10	4.5 (4.2–4.8)	4.7 (4.11–5)	0.353
Hemoglobin, g/L	115–150	134.5 (124.8–144.3)	139.5 (120.5–152)	0.655
Platelet count, × 10^9^/L	125–350	50.0 (38.8–65.8)	41.1 (26.0–50.8)	0.025
ALT, U/L	9–50	129 (61.0–214.5)	134.8 (86.6–234.5)	0.392
AST, U/L	15–40	284.7 (130.5–499.4)	402.0 (230.3–798.8)	0.041
GGT, U/L	10–60	38.9 (21.9–86.5)	98.0 (28.2–217.8)	0.032
ALP, U/L	45–125	63.5 (49.7–84.7)	83.9 (48.2–171.7)	0.134
ALB, g/L	40–55	31.1 (28.3–34.1)	29.1 (27.1–32.0)	0.058
TB, μmol/L	0–26	7.5 (5.3–11.2)	11.5 (8.7–17.7)	<0.001
LDH, U/L	120–250	695.0 (402.2–1397.4)	1393.4 (718.2–2396.0)	0.003
BUN, mmol/L	3.6–9.5	5.6 (3.7–9.9)	11.4 (5.9–17.3)	0.002
Cr, μmol/L	57–111	63.0 (50.0–83.4)	92.5 (66.7–181.6)	<0.001
TnI, μg/L	0.001–0.016	0.06 (0.02–0.15)	0.15 (0.06–0.23)	0.008

*ALT, alanine aminotransferase; AST, aspartate aminotransferase; GGT, gamma-glutamyl transpeptidase; ALP, alkaline phosphatase; ALB, albumin; LDH, lactate dehydrogenase; TB, total bilirubin; BUN, blood urea nitrogen; Cr, creatinine; TnI, cardiac troponin I.*

### Relationship Between the Combined Biomarkers and Clinical Outcome

**FIGURE 2:**
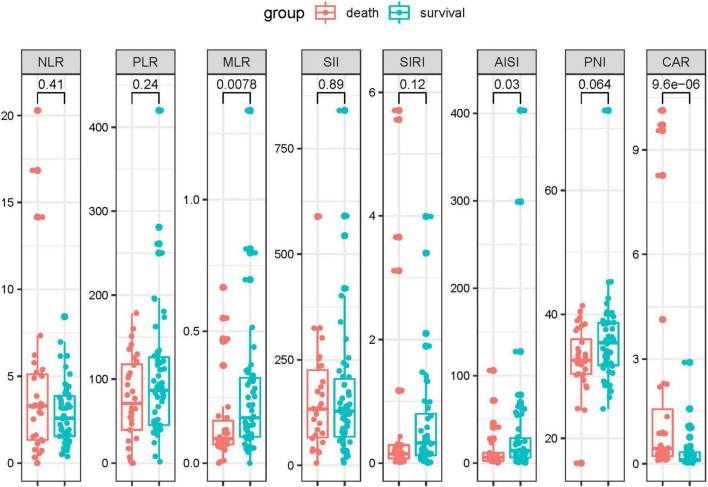
The inflammation-based biomarkers were compared between the death patients and survival patients. Statistical significance was calculated by Mann–Whitney *U* test. Data are presented as median (IQR).

The combined biomarkers reflecting inflammatory and nutrition status based on the laboratory tests were compared between the survival patients and death patients. As shown in the Figure 2, the CAR (*p* < 0.001) in the death group was remarkably higher than that of the survival group. Conversely, the AISI (*p* = 0.03) and MLR (*p* = 0.0078) were higher in the survival group compared to the death group. The other indexes, including the NLR, PLR, SII, SIRI, and PNI, did not show the differences between the two groups.

### The Dynamic Changes of the Differential Biomarkers

**FIGURE 3:**
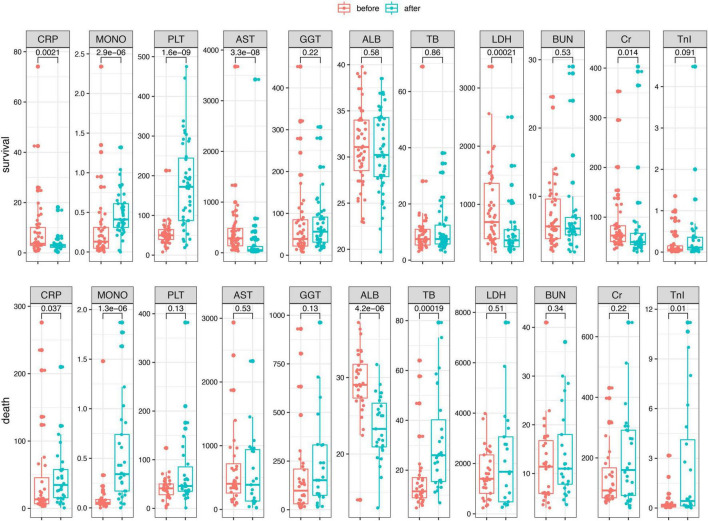
The dynamic changes of the differential biomarkers before and after treatments in the SFTS patients.

**FIGURE 4:**
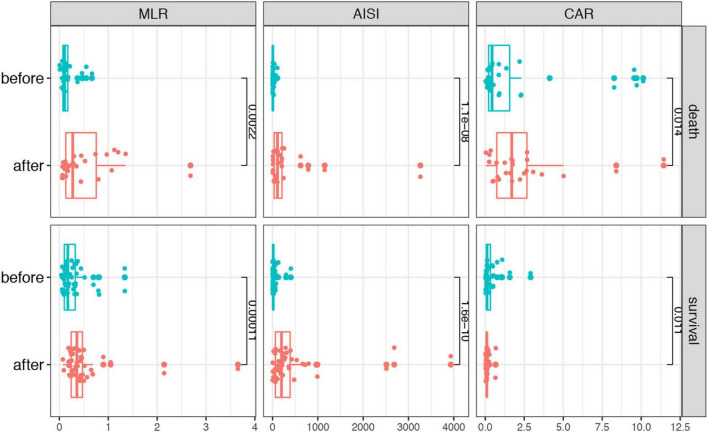
The dynamic changes of the combined biomarkers before and after treatments in the SFTS patients.

To identify if the laboratory parameters and combined biomarkers were associated the disease progression in patients with SFTS, we performed a dynamic analysis of the differential biomarkers. We further collected the last test from SFTS patients after treatments and before they left the hospital or progressed to death. Then we compared the biomarkers between the first visit and the last visit during hospitalization in the two groups. As shown in Figure 3, the CRP, AST, GGT, LDH, TB, BUN, CR, and TnI levels increased from the disease onset and remained the similar levels or even kept increasing in the death patients after treatment. And in the survival group, the CRP, AST, LDH, CR, and TnI displayed a significant trend returning to the normal range after treatment. While in the combined biomarkers (Figure 4), the MLR and AISI both had a significant increase in the death and survival group. The CAR significantly increased in the death group (*p* = 0.014) and decreased in the survival group (*p* = 0.011).

### Predictive Ability of Biomarkers for Patients With Severe Fever With Thrombocytopenia Syndrome

**FIGURE 5:**
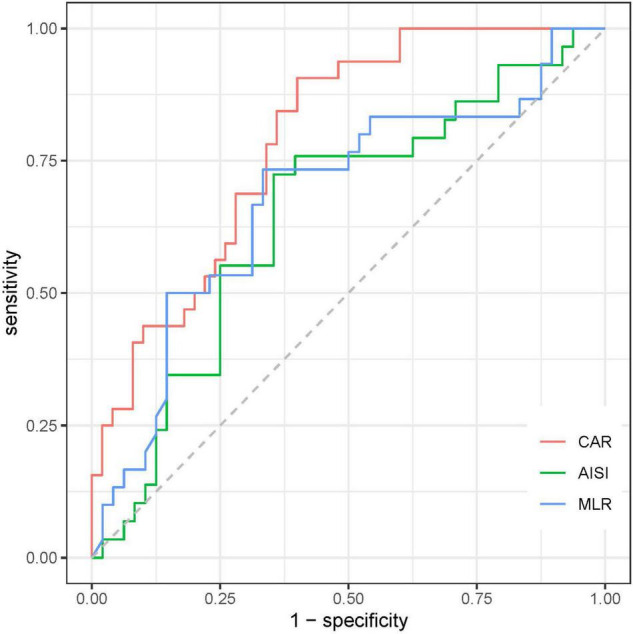
The predictive value of MLR, AISI, and CAR in the poor prognosis of SFTS patients.

To evaluate the prognostic value and calculate the best cut-off for CAR, AISI, and MLR for predicting disease prognosis in patients with SFTS, the ROC curve analysis was performed (Figure 5 and Table 2). The results showed that the areas under the curve (AUC) for survival were 0.648 (95% CI: 0.52–0.78, *p* = 0.03) for AISI, 0.68 (95% CI: 0.55–0.81, *p* = 0.008) for MLR and 0.79 (95% CI: 0.70–0.89, *p* < 0.001) for CAR, respectively. In addition, the AUC of the CAR was larger than that of MLR (*Z* = 2.088, *p* = 0.036) and AISI (*Z* = 2.331, *p* = 0.019). According to the maximum Youden index, the optimal cut-off value of CAR was 0.14 for predicting the poor prognosis, with 90.6% sensitivity and 60% specificity.

**TABLE 2:** The ROC curves and prognostic accuracy of the inflammation-based biomarkers.

	AUC	*p*-value	95% CI	Cut off point	Sensitivity (%)	Specificity (%)
CAR	0.791	<0.001	0.70–0.89	0.14	0.906	0.6
CRP	0.786	<0.001	0.70–0.88	4.66	0.906	0.6
ALB	0.625	0.058	0.50–0.75	29.26	0.7	0.562
AISI	0.648	0.03	0.52–0.78	12.26	0.604	0.759
MLR	0.68	0.008	0.55–0.81	0.13	0.667	0.733

*CAR, CRP/Albumin; CRP, C-reactive protein; ALB, albumin; AISI, AISI = (neutrophils × monocytes × platelets)/lymphocytes; PNI, albumin (g/L) + 5 × total lymphocyte counts (10^9^/L).*

**FIGURE 6:**
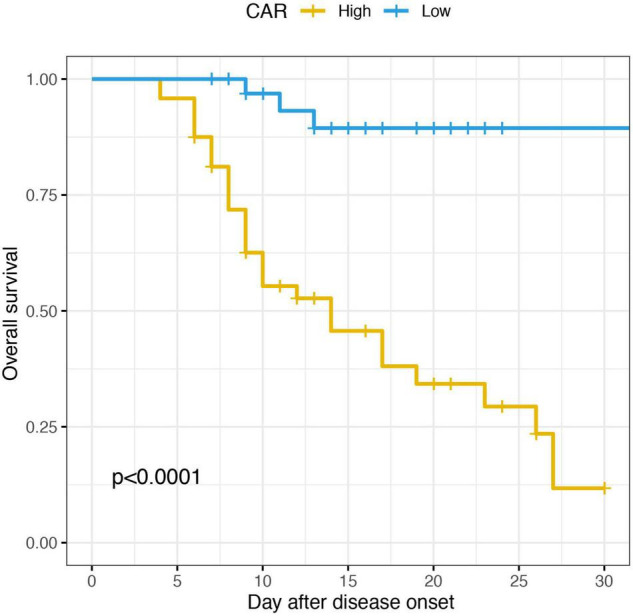
KM survival curve for overall survival in SFTS patients stratified according to CAR.

Accordingly, patients were divided into two groups in terms of the optimal cut-off value of CAR, the high group and the low group. The KM survival analysis based on the log-rank test was conducted. As shown in the Figure 6, the SFTS patients had a significant lower survival with high value of CAR.

### Univariate and Multivariate Logistic Regression Analyses of Possible Biomarkers in Patients With Severe Fever With Thrombocytopenia Syndrome

We performed univariate and multivariate logistic regression analysis to further evaluate the diagnostic value of CAR in SFTS. The differential clinical indicators were also included in the regression analysis. As shown in the Table 3, the increased levels of AST, LDH, CR, TnI, CAR and reduced PLT and monocyte counts were considered as risk factors for poor prognosis in the univariate logistic regression analysis. The multivariate logistic analysis suggested that CAR (OR = 4.8; 95% CI: 1.0–21.1; *p* = 0.043), monocyte counts (OR = 9.1; 95% CI: 1.9–44.1; *p* = 0.006) and GGT level (OR = 4.6; 95% CI: 1.0–21.5; *p* = 0.043) can be independent risk factors for predicting poor prognosis of SFTS patients.

**TABLE 3:** Logistic regression analysis of risk factors for prognosis in patients with SFTS.

Variable	Classification	Univariate analysis			Multivariate analysis		
		OR	95%CI	*p*	OR	95%CI	*p*
Age (year)	≥65 vs. <65	1.636	0.669–4.002	0.28			
Sex	male vs. female	1.083	0.446–2.632	0.86			
PLT, × 10^9^/L	≤50 vs. >50	2.769	1.046–7.332	0.04			
Monocyte count, × 10^9^/L	≤0.1 vs. >0.1	5.357	1.86–15.43	0.002	9.102	1.878–44.106	0.006
AST	≥2ULN vs. <2ULN	6.805	0.818–56.579	0.076			
GGT	≥2ULN vs. <2ULN	4.02	1.477–10.941	0.006	4.643	1.003–21.491	0.05
TB	≥ULN vs. <ULN	3.429	0.59–19.932	0.17			
LDH	≥2ULN vs. <2ULN	4.782	1.25–18.297	0.022			
Urea	≥ULN vs. <ULN	2.504	0.992–6.32	0.052			
Cr	≥ULN vs. <ULN	3.212	1.192–8.656	0.021			
TnT	≥ULN vs. <ULN	4.219	1.103–16.131	0.035			
CAR	≥0.14 vs. <0.14	5.824	2.161–15.692	<0.001	4.801	1.049–21.977	0.043
AISI	≤12.26 vs. >12.26	4.787	1.75–13.096	0.002			
MLR	≤0.13 vs. >0.13	5.015	1.841–13.663	0.002			

## Discussion

The SFTS is becoming a public health threat in China, South Korea, Japan and some other east Asian countries since 2010. SFTS mostly arises as sporadic cases in the spring and summer with a high fatality rate. The disease is characterized by abrupt onset of fever, respiratory tract or gastrointestinal symptoms, followed by a progressive decline in platelets and white blood cells (39). And the SFTSV infection is characterized by four typical periods: incubation, fever, multiple organ failure, and convalescence (39). The SFTS patients could rapidly progress to critical illness, and the average period from onset of illness to death is 9 days (40). Since there is no specific treatment and available vaccine, the symptomatic and supportive therapy are the major therapeutic methods. There is an urgent need of the early biomarkers to identify SFTS patients at higher risk of death, and these patients need more attention of early diagnosis and aggressive treatment.

The combined indexes based on the complete blood count and inflammatory indicators have been widely used as inflammatory biomarkers. These parameters could be simply and inexpensively obtained. Therefore, the combined biomarkers are recommended to assist in testing inflammatory processes, predicting the disease prognosis and risk stratification. In our study, we evaluated the clinical and prognostic roles of various inflammatory and nutritional indexes for SFTS, including the NLR, PLR, MLR, AISI, SII, SIRI, PNI, and CAR. Of them, CAR is higher in patients with fatal disease than in those with survival outcome. While the value of AISI and MLR was higher in the survival group compared to the death group. The logistic regression analysis discovered that CAR was an independent prognostic biomarker for poor prognosis in patients with SFTS. The CAR also exhibited a more robust risk assessment value in the ROC curve analysis. And in the dynamic analysis, the CAR kept increasing in the death group while gradually decreased in the survival group after receiving treatments during hospitalization. Eventually, CAR was considered as a promising early predictive biomarker of evaluating the SFTS patients.

C-reactive protein to albumin ratio is integrated by the two indexes, CRP and ALB. ALB is mainly synthesized by liver and is the most abundant circulating protein in the blood. It is the main determinant of plasma oncotic pressure, and plays a pivotal role in modulating the distribution of fluids between body compartments (41). The plasma ALB level might fall during the periods of stress, trauma, injury and infection, due to the redistribution from the intravascular to extravascular space, decreased synthesis and increased catabolism. In addition, ALB could reflect the nutritional status of body and contribute to the transportation of multiple substances. In our study, lower ALB level was also observed in the SFTS patients with poor clinical outcome compared to the survivors. This is possibly affected by the severe infection and multiple organ dysfunction resulted from the SFTSV. CRP exhibits elevated expression during inflammatory conditions such as rheumatoid arthritis, some cardiovascular diseases, and infection (42). As an acute-phase protein, the plasma concentration of CRP deviates by at least 25% during inflammatory disorders (43). While in our study, the CRP level was significant higher in the death group compared to the survival group which is consistent with the previous studies (44).

The association between inflammation and malnutrition is very close and complicated. The inflammation could result in malnutrition, and the malnutrition in turn could impact the development of inflammation. As a combined index, the CAR is becoming a promising prognostic biomarker in patients with various diseases, including the tumors (45–47), infectious diseases (48, 49), autoimmune diseases (31, 50) and some other diseases (51). However, no research about whether CAR can evaluate the prognosis of patients with SFTS has been reported so far. Our study showed that the CAR is able to be used as an independent risk factor with good predictive potential for a poor prognosis of SFTS patients. It could provide the physicians the information for predicting the early risk stratification of disease progression of SFTS patients and guide them to monitor and treat higher-risk patients more aggressively, which may help to improve clinical prognosis.

However, there were also limitations in our study. Firstly, this was a retrospective, single-center study with a relatively small cohort size and the number of death cases was also small which might lead to a possible statistical bias. Secondly, the observational endpoint of our study was defined as the in-hospital death lacking of further follow-up. Hence, a multi-center study with a larger cohort size and clinical follow-up visits is urgent in the future.

## Conclusion

In conclusion, our study first found the CAR was significantly associated with the mortality of SFTS patients. Higher CAR was demonstrated to be an independent risk factor for death in patients with SFTS and could be used to predict the poor prognosis of SFTS.

## Data Availability Statement

The original contributions presented in the study are included in the article/supplementary material, further inquiries can be directed to the corresponding authors.

## Ethics Statement

The studies involving human participants were reviewed and approved by the Research Ethics Committee of the Second Affiliated Hospital of Dalian Medical University. The Ethics Committee waived the requirement of written informed consent for participation.

## Author Contributions

YL and RL designed the study. HY and CX collected the data from the electronic record system. XY and RL performed the data analyses and interpreted the data. XY and YL contributed to the writing of the manuscript. All authors reviewed the manuscript and approved the final version.

## Conflict of Interest

The authors declare that the research was conducted in the absence of any commercial or financial relationships that could be construed as a potential conflict of interest.

## Publisher’s Note

All claims expressed in this article are solely those of the authors and do not necessarily represent those of their affiliated organizations, or those of the publisher, the editors and the reviewers. Any product that may be evaluated in this article, or claim that may be made by its manufacturer, is not guaranteed or endorsed by the publisher.
